# Sodium nitroprusside prevents the detrimental effects of glucose on the neurovascular unit and behaviour in zebrafish

**DOI:** 10.1242/dmm.039867

**Published:** 2019-09-01

**Authors:** Karishma Chhabria, Avgoustinos Vouros, Caroline Gray, Ryan B. MacDonald, Zhen Jiang, Robert Neil Wilkinson, Karen Plant, Eleni Vasilaki, Clare Howarth, Timothy J. A. Chico

**Affiliations:** 1 Neuroimaging in Cardiovascular Disease (NICAD) Network, University of Sheffield, Sheffield, S10 2TN, UK; 2 Department of Infection, Immunity and Cardiovascular Disease, University of Sheffield Medical School, Beech Hill Road, Sheffield, S10 2RX, UK; 3 The Bateson Centre, Firth Court, University of Sheffield, Western Bank, Sheffield, S10 2TN, UK; 4 Department of Computer Science, University of Sheffield, Portobello, Sheffield, S1 4DP, UK; 5 Department of Psychology, University of Sheffield, Cathedral Court, 1 Vicar Lane, Sheffield, S1 2LT, UK

**Keywords:** Nitric oxide, NO donor, Hyperglycemia, Diabetes, Neurovascular coupling

## Abstract

Diabetes is associated with dysfunction of the neurovascular unit, although the mechanisms of this are incompletely understood and currently no treatment exists to prevent these negative effects. We previously found that the nitric oxide (NO) donor sodium nitroprusside (SNP) prevents the detrimental effect of glucose on neurovascular coupling in zebrafish. We therefore sought to establish the wider effects of glucose exposure on both the neurovascular unit and on behaviour in zebrafish, and the ability of SNP to prevent these. We incubated 4-days post-fertilisation (dpf) zebrafish embryos in 20 mM glucose or mannitol for 5 days until 9 dpf, with or without 0.1 mM SNP co-treatment for 24 h (8-9 dpf), and quantified vascular NO reactivity, vascular mural cell number, expression of a *klf2a* reporter, glial fibrillary acidic protein (GFAP) and transient receptor potential cation channel subfamily V member 4 (TRPV4), as well as spontaneous neuronal activation at 9 dpf, all in the optic tectum. We also assessed the effect on light/dark preference and locomotory characteristics during free-swimming studies. We find that glucose exposure significantly reduced NO reactivity, *klf2a* reporter expression, vascular mural cell number and TRPV4 expression, while significantly increasing spontaneous neuronal activation and GFAP expression (all in the optic tectum). Furthermore, when we examined larval behaviour, we found that glucose exposure significantly altered light/dark preference and high and low speed locomotion while in light. Co-treatment with SNP reversed all these molecular and behavioural effects of glucose exposure. Our findings comprehensively describe the negative effects of glucose exposure on the vascular anatomy, molecular phenotype and function of the optic tectum, and on whole-organism behaviour. We also show that SNP or other NO donors may represent a therapeutic strategy to ameliorate the complications of diabetes on the neurovascular unit.

This article has an associated First Person interview with the first author of the paper.

## Abstract (precis)

**Summary:** Diabetes is associated with vascular and neurological impairments. The authors show that an NO donor ameliorates the glucose-exposure-induced dysfunction in the tectal neurovascular unit and whole-organism behaviour.

## INTRODUCTION

The prevalence of diabetes has quadrupled in the previous two decades, incurring an enormous burden of morbidity and healthcare expenditure worldwide (Zhang et al., 2010; Scully, 2012). Diabetes is a risk factor for both macrovascular (such as myocardial infarction and stroke) and microvascular (causing renal impairment and retinopathy) disease (Chase et al., 1989; Jørgensen et al., 1994; Miettinen et al., 1998; Stratton et al., 2000). Diabetes is also associated with neurological disorders, including cognitive impairment (dementia) (Stewart and Liolitsa, 1999; Areosa and Grimley, 2002; MacKnight et al., 2002; Ciudin et al., 2017; Simó et al., 2017; Groeneveld et al., 2018). The mechanisms underlying this association are incompletely understood, and no specific therapies have been identified to prevent or reverse the effects of diabetes on neurological function.

Both diabetes and neurological diseases are associated with dysfunction of the neurovascular unit (NVU) (Zlokovic, 2010; Mogi and Horiuchi, 2011; Gardner and Davila, 2017). The NVU comprises neurons, astrocytes, myocytes, pericytes, endothelial cells (ECs) and extracellular matrix. Interactions between these ensures that neuronal energy demands are met through increased local blood flow via neurovascular coupling (NVC) (Roy and Sherrington, 1890; Attwell et al., 2010). Recent evidence suggests that ECs are crucial to NVU function (Toth et al., 2015; Guerra et al., 2018) as they release vasoactive substances such as nitric oxide (NO) (Ignarro et al., 1987; Palmer et al., 1987). NO production is regulated by various endothelial genes, including the Kruppel-like family of transcription factors (KLFs) (Gracia-Sancho et al., 2011), particularly KLF2, which is regulated by changes in flow and inflammation (Dekker et al., 2002; SenBanerjee et al., 2004). ECs share a common basement membrane with pericytes that aid EC development (Gerhardt and Betsholtz, 2003). Pericyte coverage, essential to both blood-brain barrier (BBB) integrity and NVC, is affected in various neuropathologies (Tilton et al., 1985; Frank et al., 1990; Peppiatt et al., 2006; Pfister et al., 2008; Vates et al., 2010; Sagare et al., 2013; Hall et al., 2014). Astrocytes are the predominant glial cell in the brain and perform several functions, including release of vasoactive factors (Zonta et al., 2003; Takano et al., 2006). Astrocytes also express glutamine synthetase (GS), an enzyme involved in the recycling of glutamate released by active neurons (Bergles et al., 1999; Bringmann et al., 2013). Mammalian studies show that astrocytes sense changes in vascular tone through activation of the mechanosensor TRPV4 (transient receptor potential cation channel subfamily V member 4) (Filosa et al., 2013), which is also expressed in ECs. Together, all these cell types maintain the functional NVU.

The zebrafish is increasingly used as a model of human disease (Dooley and Zon, 2000; Lieschke and Currie, 2007). This has a number of advantages over existing mammalian models, particularly ease of *in vivo* cellular imaging and the ability to test the effect of drugs by immersion. Although most zebrafish studies attempting to model human disease have examined the anatomical or molecular effects of genetic or other manipulation (Patton and Zon, 2001; Lieschke and Currie, 2007), a range of behavioural assays have been applied to study more complex ‘whole organism’ phenotypes, such as memory or aggression (Blaser and Gerlai, 2006; Oliveira et al., 2011). The zebrafish has previously been used as a model of diabetes, either by exposure to medium containing glucose (Capiotti et al., 2014), genetic manipulation (Kimmel et al., 2015) or ablation of the β-cells of the pancreas (Pisharath et al., 2007).

We recently established a novel zebrafish larval model of NVC in which incubation of larvae in 20 mM glucose impaired NVC. We found that the NO donor sodium nitroprusside (SNP) rescued this effect (Chhabria et al., 2018), although the mechanism for this is unclear. We therefore wish to better understand the mechanism and consequences of NVU dysfunction induced by glucose. In the present study, we have now examined the effect of glucose exposure on: NO production in the NVU, *klf2a* expression, mural cell number, glial fibrillary acidic protein (GFAP) expression, TRPV4 expression, spontaneous neuronal activation, light/dark preference and larval locomotory behaviour (exploratory and thigmotactic). We find that glucose exposure affects all these aspects of NVU function and behaviour, and that co-treatment with SNP completely prevents all the detrimental effects of glucose exposure. Our findings provide insight into the effect of hyperglycemia on NVU function and further support for the possibility that NO donors represent plausible drug candidates to ameliorate the detrimental effects of hyperglycemia.

## RESULTS

### SNP co-treatment prevents the reduced vascular NO reactivity caused by glucose exposure

**Fig. 1.**
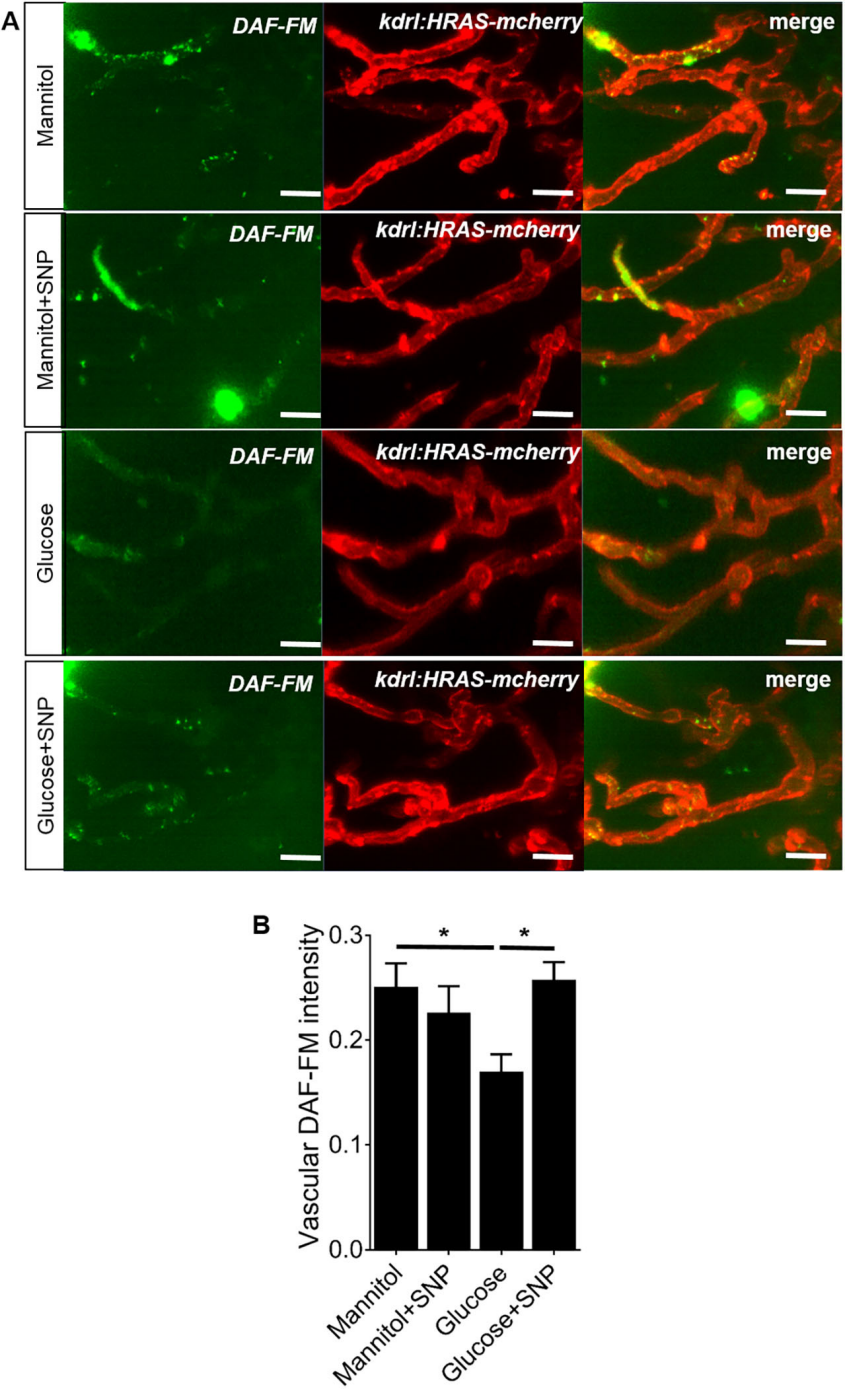
**Effect of mannitol/glucose treatment with/without** **sodium nitroprusside** **(SNP) on** **nitric oxide** **(NO) reactivity, quantified by intensity of DAF-FM staining.** (A) Representative micrographs of tectal vessels showing separate and merged channels (green: DAF-FM staining; red: *kdrl:HRAS-mCherry*) for 20 mM mannitol- or glucose-exposed larvae co-treated with or without SNP. Scale bars: 20 µm. (B) Quantification of DAF-FM intensity in the tectal vessels (*n*=25, 24, 27 and 24 larvae for mannitol, mannitol+SNP, glucose and glucose+SNP, respectively). Data are mean±s.e.m. *P<0.05.

Studies with diabetic patients have shown reduced bioavailability of NO in ECs (Williams et al., 1996; Pieper, 1998). We therefore first examined whether glucose exposure reduces NO availability in the cerebral vessels. We used diaminofluorescein-FM (DAF-FM) staining to visualise NO reactivity in 9 days post-fertilisation (dpf) zebrafish embryos exposed to 20 mM glucose or mannitol (as osmotic control) with or without co-treatment with 0.1 mM SNP. Representative micrographs of the vessels in the left optic tectum are shown in Fig. 1A. We observed variable levels of NO reactivity that colocalised with the *kdrl:HRAS-mCherry* endothelial reporter in animals treated with mannitol. Co-treatment with mannitol and the NO donor, SNP, did not alter the intensity of vascular NO reactivity compared to treatment with mannitol alone (Fig. 1). Exposure to glucose significantly reduced vascular NO reactivity in the tectal vessels (Fig. 1), in keeping with data from other models (Pieper, 1998; Du et al., 2001). Co-treatment with SNP prevented this reduction in vascular NO reactivity (Fig. 1).

### SNP co-treatment prevents the reduced endothelial *klf2a* expression caused by glucose exposure

**Fig. 2.**
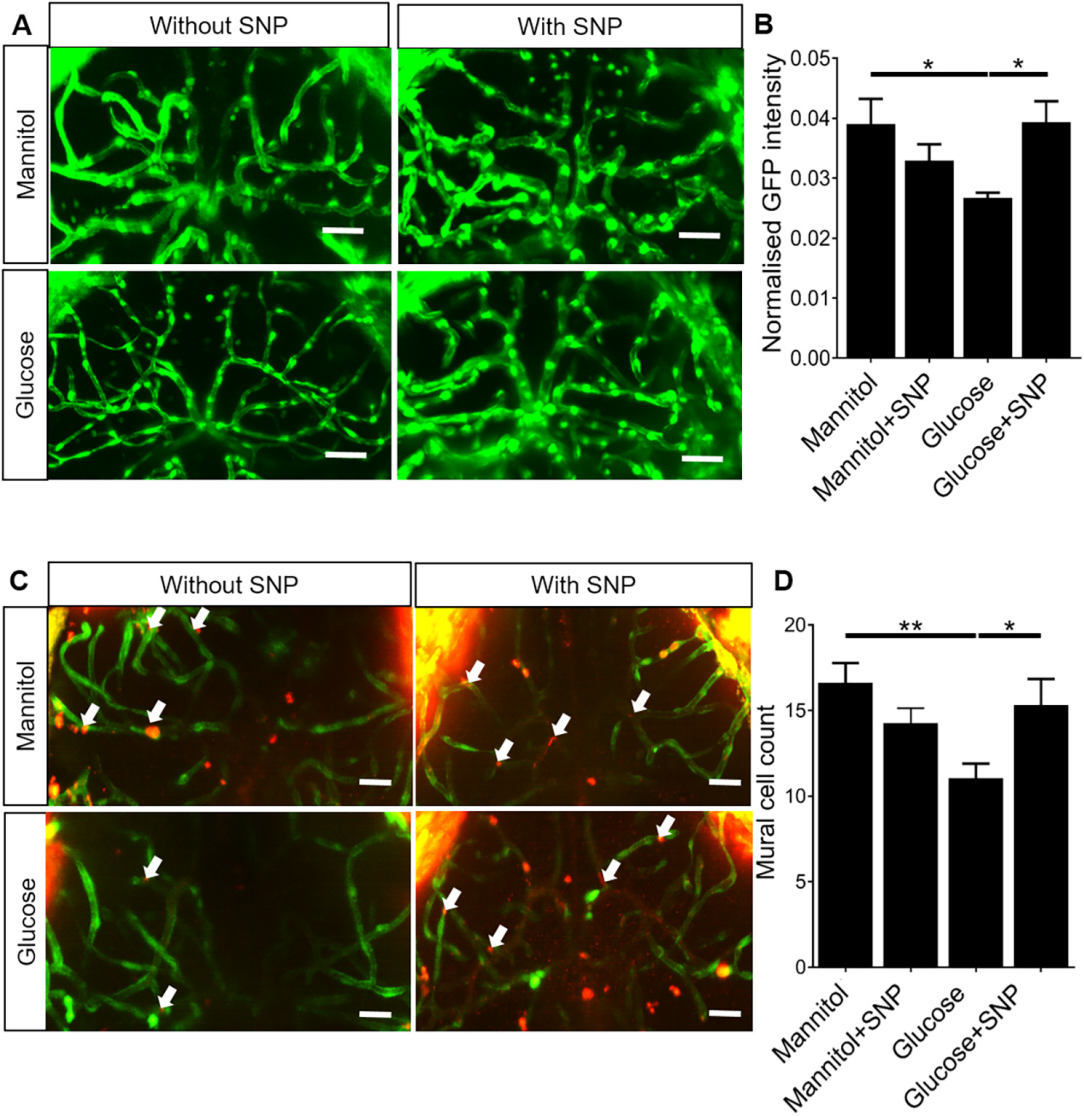
**Effect of mannitol/glucose treatment with/without** **sodium nitroprusside** **(SNP)** **on** ***klf2a:GFP* expression and number of mural cells** **on the** **tectal vessels.** (A) Representative micrographs of tectal vessels in *Tg(klf2a:GFP)* exposed to 20 mM mannitol or glucose co-treated with or without SNP. Scale bars: 20 µm. (B) Quantification of *klf2a:GFP* intensity in the tectal vessels (*n*=26 larvae/group). (C) Representative micrographs of tectal vessels showing separate and merged channels (green: *fli1:GFF:UAS:GCaMP6*; red: *sm222ab:nls-mCherry ^sh480^*) for 20 mM mannitol- or glucose-exposed larvae co-treated with or without SNP. Scale bars: 20 µm. Arrows indicate mural cell nuclei. (D) Quantification of the number of *sm22ab:nls-mCherry^sh480^* nuclei on the tectal vessels for 20 mM mannitol- or glucose-exposed larvae co-treated with or without SNP (*n*=28 larvae/group). Data are mean±s.e.m. *P<0.05, **P<0.01.

The shear-stress-responsive transcription factor *klf2a* is protective against vascular disease (Dekker et al., 2002, 2005; Chiu et al., 2009). To determine whether glucose exposure affects endothelial shear-stress sensing and *klf2a* expression, we quantified the intensity of a *Tg(klf2a:GFP)* reporter in the cerebral vessels of zebrafish with glucose or mannitol with or without SNP co-treatment. Representative micrographs are shown in Fig. 2A. Glucose exposure significantly reduced intensity of the *klf2a:GFP* reporter compared to mannitol alone (Fig. 2B). Although SNP co-treatment with mannitol had no effect compared with mannitol alone (Fig. 2B), SNP co-treatment prevented the glucose-induced reduction in *klf2a* reporter expression.

### SNP co-treatment prevents the reduced number of vascular mural cells on the tectal vessels caused by glucose exposure

NO is required for mural cell function and can evoke hyperpolarization in mural cells (including pericyte and smooth muscle cell), causing vasodilation (Sakagami et al., 2001; Lee et al., 2005). Mural cell loss is a feature of diabetes (Pfister et al., 2008), but no therapy has been shown to reverse this. We therefore examined the effect of glucose on vascular mural cells. We used an *sm22ab:nls-mCherry* reporter to quantify the number of vascular mural cells present in the optic tectum. Representative micrographs are shown in Fig. 2C. Glucose exposure induced a significant reduction in the number of vascular mural cells compared with either mannitol or mannitol plus SNP (Fig. 2D). Co-treatment with SNP prevented the reduction of vascular mural cells induced by glucose exposure.

### SNP co-treatment prevents the increased GFAP expression in the optic tectum caused by glucose exposure

**Fig. 3.**
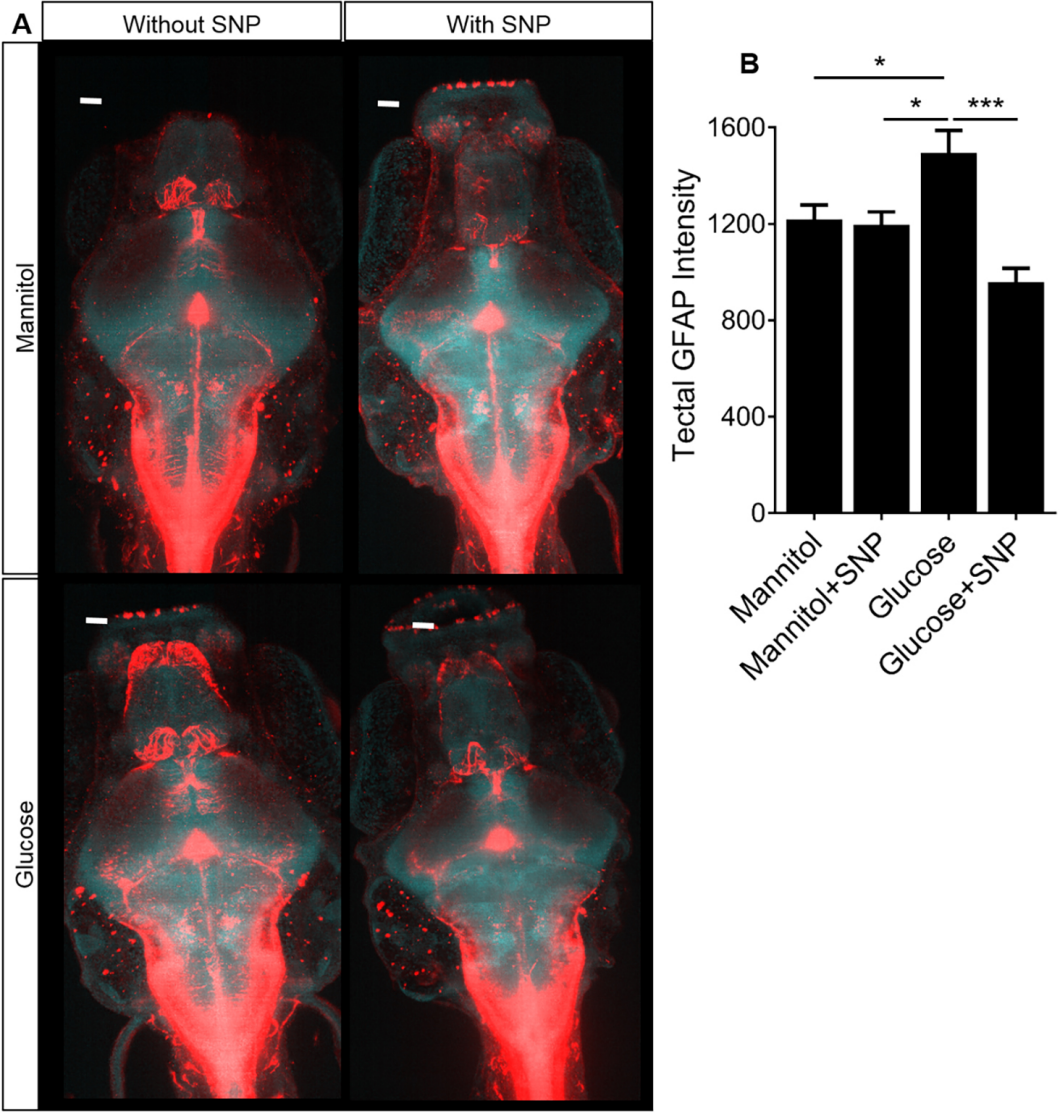
**Effect of mannitol/glucose treatment with/without** **sodium nitroprusside** **(SNP) on** **glial fibrillary acidic protein** **(GFAP) expression.** (A) Representative micrographs showing the effect of mannitol/glucose exposure with or without SNP treatment on GFAP expression (red channel represents GFAP; blue channel represents DAPI). Scale bars: 20 µm. (B) Quantification of GFAP expression in the optic tectum (*n*=16, 12, 18 and 20 larvae for mannitol, mannitol+SNP, glucose and glucose+SNP, respectively). Data are mean±s.e.m. *P<0.05, ***P<0.001.

Glial cells play major roles in the maintenance of the BBB and NVU function (Janzer and Raff, 1987; Prat et al., 2001). Experimental studies have shown overexpression of GFAP (termed astrogliosis) in response to both hyperglycemia and type 1 diabetes (Coleman et al., 2004). We thus examined the effect of glucose or mannitol with or without SNP on GFAP expression. Representative micrographs of whole-mount 9 dpf zebrafish are shown in Fig. 3A. Glucose exposure increased GFAP expression compared to mannitol treatment (Fig. 3B), in keeping with astrogliosis in other diabetic models (Coleman et al., 2004). This was prevented by co-treatment with SNP (Fig. 3B).

### SNP co-treatment prevents the reduced expression of TRPV4 in the optic tectum caused by glucose exposure

**Fig. 4.**
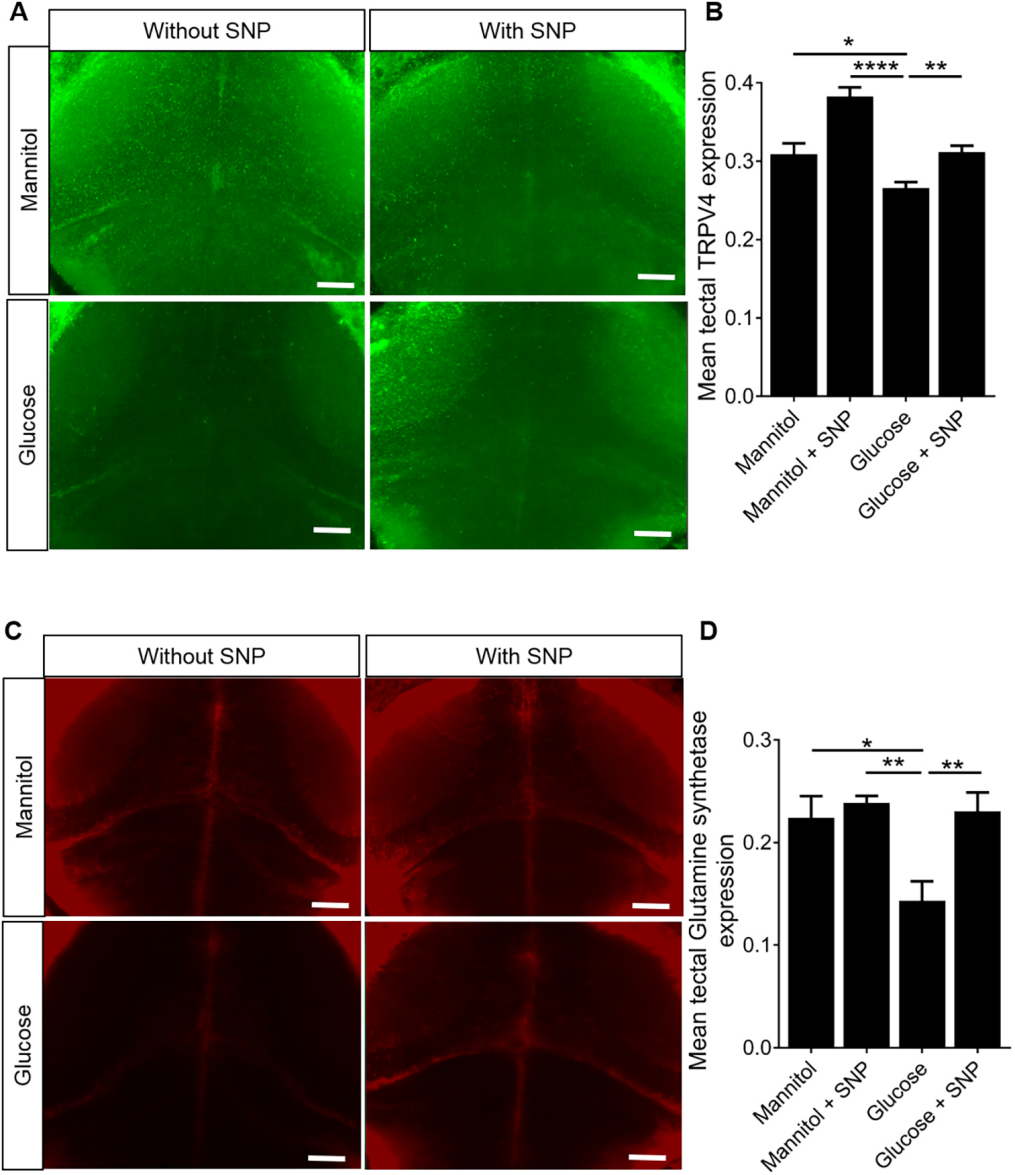
**Effect of mannitol/glucose treatment** **with/without** **sodium nitroprusside** **(SNP) on TRPV4 and glutamine synthetase** **(GS).** (A) Representative micrographs of optic tectum showing the effect of mannitol/glucose treatment with/without SNP treatment on the expression of TRPV4. Scale bars: 20 µm. (B) Quantification of the TRPV4 intensity in the optic tectum in a fixed volume of the tissue (*n*=21, 20, 24 and 26 larvae for mannitol, mannitol+SNP, glucose and glucose+SNP, respectively). (C) Representative micrographs of optic tectum showing the effect of mannitol/glucose treatment with/without SNP treatment on the expression of GS. Scale bars: 20 µm. (D) Quantification of the GS intensity in the optic tectum in a fixed volume of tissue (*n*=21, 20, 24 and 26 larvae for mannitol, mannitol+SNP, glucose and glucose+SNP, respectively). Data are mean±s.e.m. *P<0.05, **P<0.01, ****P<0.0001.

Since hyperglycemia downregulates TRPV4 in the ECs of the retinal microvasculature (Monaghan et al., 2015), we investigated TRPV4 expression by immunohistochemistry (IHC) in 9 dpf zebrafish larvae exposed to glucose or mannitol with or without SNP treatment. We performed IHC for TRPV4 to first compare expression patterns in the optic tectum. Representative micrographs are shown in Fig. 4A. Glucose exposure decreased tectal TRPV4 expression (which includes radial glial, endothelial or neuronal expression of TRPV4) compared to mannitol exposure. Co-treatment with SNP prevented the effect of glucose on TRPV4 expression (Fig. 4B).

### SNP co-treatment prevents the reduced expression of glutamine synthetase in the optic tectum caused by glucose exposure

GS, a glial-specific enzyme involved in recycling of extracellular/extrasynaptic glutamate to glutamine, is reduced in neurological disorders and diabetes (Lieth et al., 2000; Burbaeva et al., 2003). We therefore examined expression of GS in the optic tectum. Glucose exposure induced a significant reduction in GS expression, which was prevented by SNP co-treatment (Fig. 4C,D).

### SNP co-treatment prevents the increased frequency of spontaneous neuronal calcium transients caused by glucose exposure

**Fig. 5.**
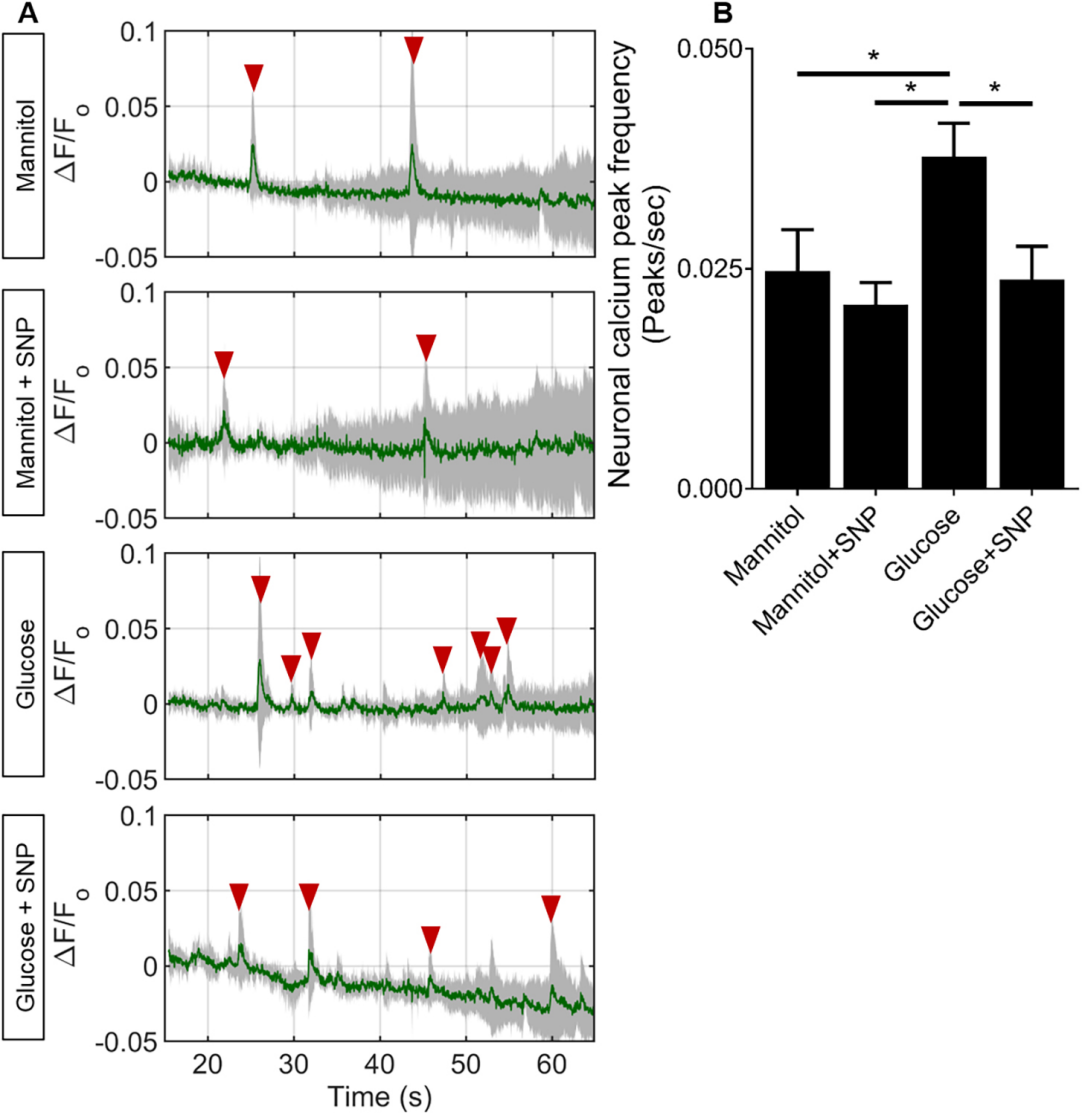
**Effect of mannitol/glucose treatment with/without** **sodium nitroprusside** **(SNP) on frequency of spontaneous neuronal calcium transients.** (A) Time series of neuronal activation (Δ*F/F*_o_) in zebrafish (5 larvae/group) exposed to mannitol, mannitol+SNP, glucose or glucose+SNP. Arrowheads indicate the detected peaks. (B) Quantification of frequency of neuronal calcium transients for each of the groups (*n*=28 larvae/group). Data are mean± s.d. in A (mean shown in green, s.d. shown in grey) and mean±s.e.m. in B. *P<0.05.

The previous results showed that glucose exposure affects both the anatomy of the NVU (vascular mural cell loss) and induces dysregulation of gene expression, including a reduction of GS, which might be expected to cause neuronal hyperexcitability. We therefore quantified neuronal activity in our model. Representative time series of spontaneous neuronal activation (quantified as *ΔF/F*_o_) in larvae exposed to mannitol or glucose with or without SNP are shown in Fig. 5A. Glucose exposure increased neuronal activation compared to mannitol exposure (Fig. 5B). Co-treatment with SNP prevented this glucose-induced increase in neuronal activation (Fig. 5B), suggesting that diabetes-induced neuronal hyperexcitability may be mediated via reduced NO bioavailability.

### SNP co-treatment prevents the altered light/dark preference and locomotion caused by glucose exposure

**Fig. 6.**
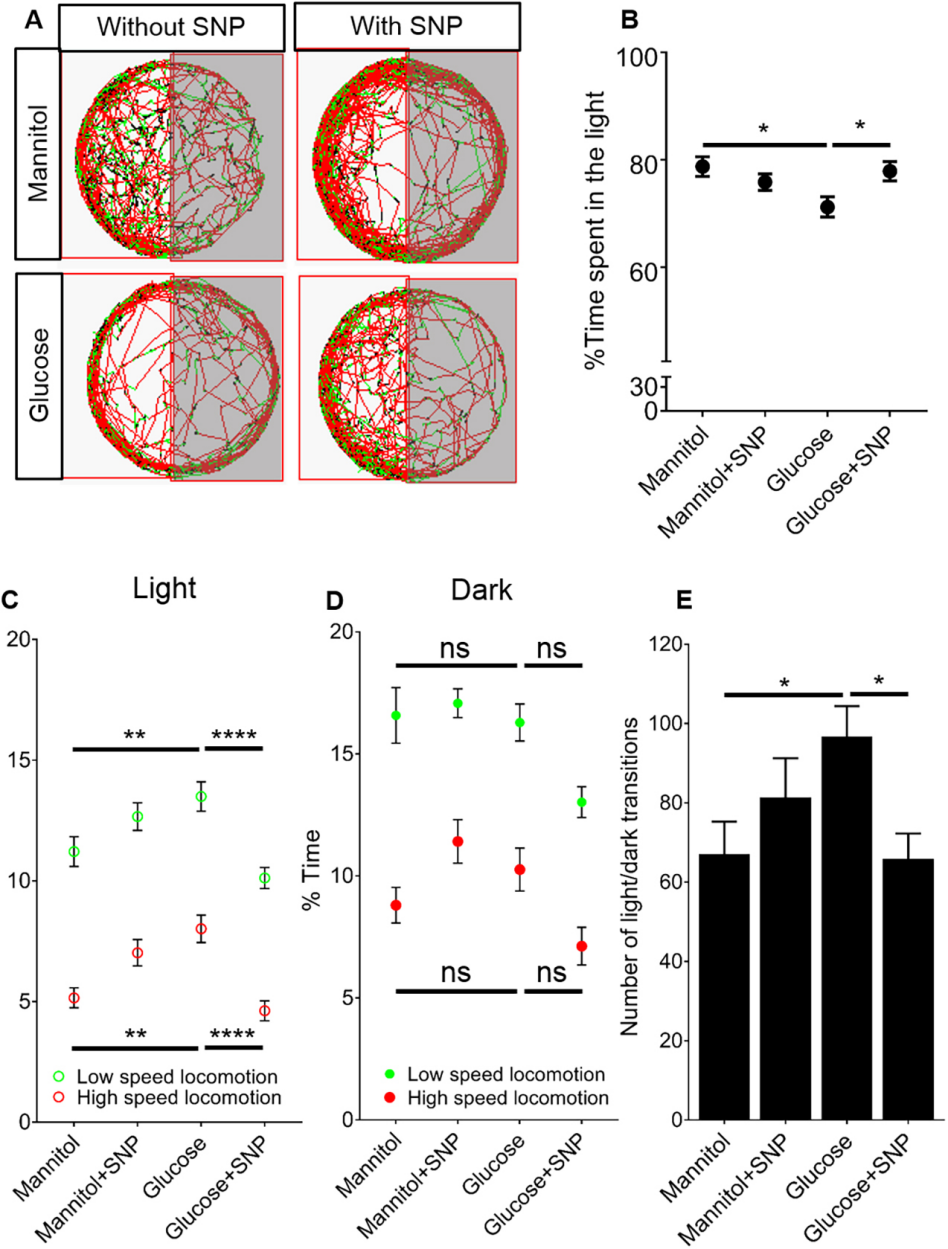
**Effect of mannitol/glucose treatment with/without** **sodium nitroprusside** **(SNP) on larval zebrafish behaviour.** (A) Representative trajectories of 9 dpf zebrafish moving in half-darkened wells (of a 12-well plate) as tracked by Viewpoint software for mannitol or glucose with or without SNP treatment. Red trajectories represent high speed (>6.4 mm/s), green low speed (3.3-6.3 mm/s), and black inactive (<3.3 mm/s). (B) Percentage of time spent in light region of the well by larvae (*n*=50, 45, 44 and 56 larvae for mannitol, mannitol+SNP, glucose and glucose+SNP, respectively). (C) Quantification of percentage time spent in low and high speed locomotion in the light region by the same animals as in B. (D) Quantification of percentage time spent in low and high speed locomotion in the dark region by the same animals as B and C. (E) Quantification of number of transitions into the light/dark regions for same larvae as in B-D. Data are mean±s.e.m. *P<0.05, **P<0.01 and ****P<0.0001.

Although the previous data clearly demonstrate molecular and functional defects of the NVU induced by glucose, if such disturbances are clinically relevant, they would be expected to manifest in overt behavioural or neurological consequences. We therefore assessed the effect of glucose exposure on free-swimming behaviour in zebrafish larvae to attempt to identify behavioural consequences of glucose exposure. We first examined the effect of glucose exposure on light/dark preference. Figure 6A shows the trajectories of representative zebrafish larvae for a period of 1 h in each treatment group. Red and green paths indicate high and low speed locomotion, respectively. We tested light/dark preference using the percentage of time spent in the light or dark side of the well. Mannitol-exposed larvae at 9 dpf showed a preference for light, spending ∼80% of time in the light. This was significantly reduced by glucose exposure (Fig. 6B). However, co-treatment with SNP prevented the effect of glucose on light/dark preference (Fig. 6B).

To further characterise larval behaviour, we quantified the time spent in high speed (>6.4 mm/s), low speed (3.3-6.3 mm/s) and inactive (<3.3 mm/s) locomotion in the light or dark side of the wells (Fig. 6C,D). Glucose exposure significantly increased the time larvae spent in both low and high speed locomotion while in the light, and SNP prevented this effect (Fig. 6C). In contrast, glucose did not induce any significant difference in the amount of time spent in either high or low speed locomotion while in the dark side of the well (Fig. 6D). We quantified the number of transitions between light and dark areas of the well and found that glucose exposure induced a significant increase in the number of transitions, and that this increase was prevented by co-treatment with SNP (Fig. 6E).

**Fig. 7.**
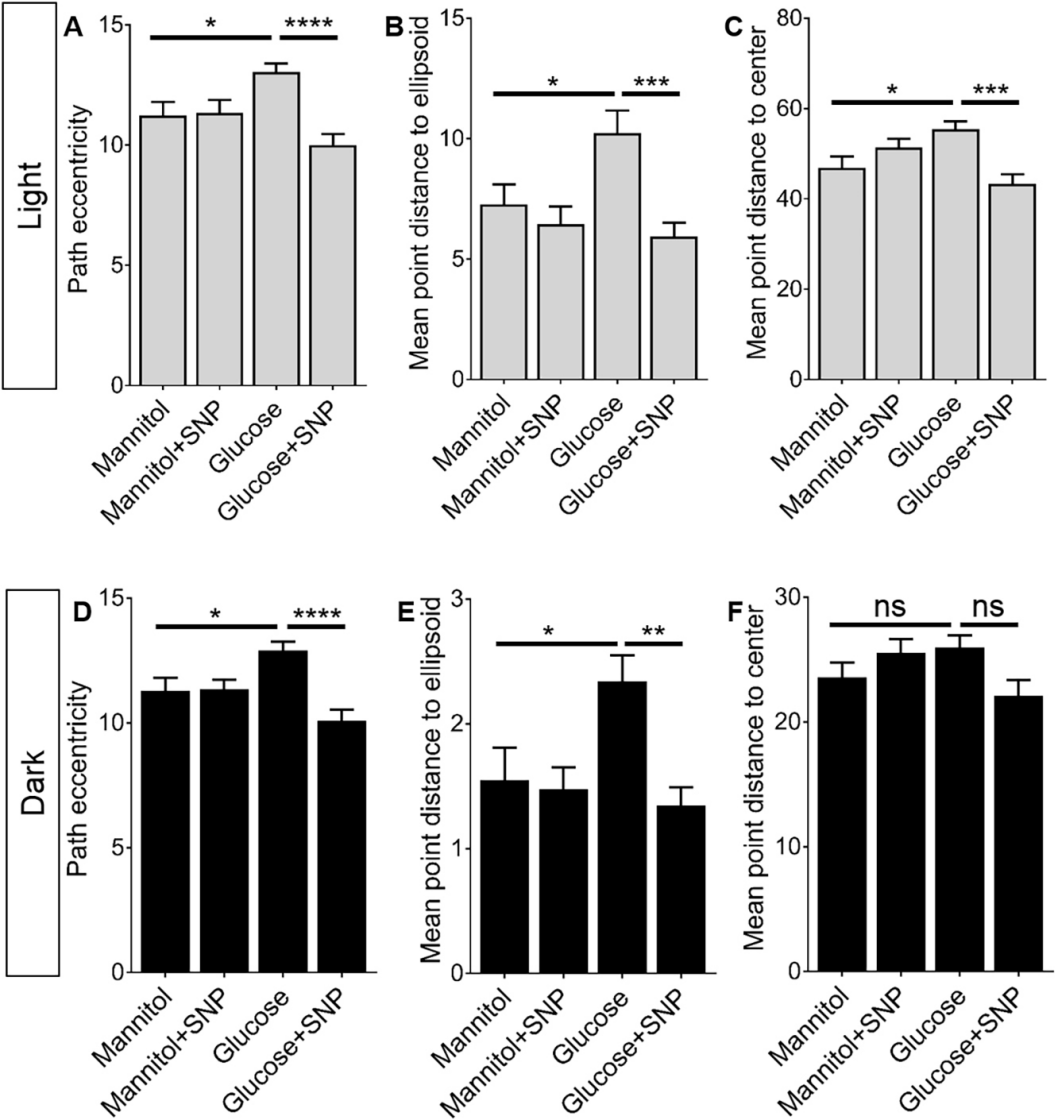
**Effect of mannitol/glucose treatment with/without** **sodium nitroprusside** **(SNP) on various features of zebrafish locomotion.** Quantification of mean frequency of path eccentricities (A,D), mean point distance to ellipsoid (MPDE; B,E) and mean point distance to centre (MPDC; C,F) (*n*=50, 45, 44 and 56 larvae for mannitol, mannitol+SNP, glucose and glucose+SNP, respectively) in (A-C) light and (D-F) dark regions of the well. Data are mean±s.e.m. *P<0.05, **P<0.01, ***P<0.001, ****P<0.0001.

We next investigated three further features of larval zebrafish locomotion; path eccentricity, mean point distance to ellipsoid (MPDE) and mean point distance to centre (MPDC) (Fig. S1) in the light and dark sides of the wells. Glucose induced significant increases in both exploratory behaviour (as measured by path eccentricities and MPDE) and thigmotaxis (MPDC) in the light side of the well; these increases were prevented by SNP (Fig. 7A-C). When we quantified these behaviours in the dark side of the well, we saw a similar significant increase in path eccentricity and MPDE but not in MPDC (Fig. 7D-F). When we performed the same analysis in blocks of 15 min, we found that these effects were apparent at different times during the hour-long observation period. Notably, the significantly higher proportion of time spent in both high and low speed locomotion in light conditions for zebrafish exposed to glucose was only observed early in the period of observation (Fig. S1), with no differences at 45 or 60 min. In contrast, the increases in light/dark transitions, path eccentricity, MPDE and MPDC in glucose-exposed zebrafish were spread more evenly across the entire observation period (Figs S1, S2). Again, SNP prevented the effect of glucose on these aspects of behaviour.

## DISCUSSION

Here, we describe a comprehensive molecular, anatomical, functional and behavioural study of the effect of glucose exposure on the NVU in zebrafish. The zebrafish model has a range of advantages, coupling simplicity, speed and cost-effectiveness with sophisticated *in vivo* imaging in a whole-organism setting. We show that a relatively short (5 days) exposure to 20 mM glucose [a concentration seen in the blood of humans with poorly controlled diabetes (Amiel et al., 1988; Boyle et al., 1988)] affects every constituent cell type of the NVU that we examined.

Glucose exposure impaired both vascular NO production and vascular mural cell number, which builds on our previous work showing that tectal endothelial patterning is impaired by glucose exposure (Chhabria et al., 2018). These effects were also associated with reduced endothelial *klf2a* expression, which is known to promote vascular inflammatory gene expression, thrombosis and atherosclerosis (SenBanerjee et al., 2004; Lin et al., 2005). Developmental studies in *klf2a^−/−^* mice have shown reduced vascular mural cell recruitment, suggesting the role of *klf2a* in maintaining endothelial-mural cell interactions (Wu et al., 2008; Fukuhara et al., 2009; Gaengel et al., 2009). Abnormal mural cell recruitment or migration is associated with various microangiopathies and is commonly observed in diabetes (Hammes et al., 2002; Gaengel et al., 2009).

In addition to the negative effects of glucose on the vascular component of the NVU, we found that glucose exposure induced upregulation of GFAP, indicating astrogliosis, with concomitant reductions in GS and TRPV4. In both rodents and zebrafish, TRPV4 channels are expressed on astrocytes, neurons and ECs (Vriens et al., 2005; Benfenati et al., 2007; Grant et al., 2007; Mangos et al., 2007; Marrelli et al., 2007; Amato et al., 2012). Interestingly, studies have shown that TRPV4 in ECs can regulate endothelial NO synthase (eNOS) (Sukumaran et al., 2013) and the presence of a feedback loop from eNOS to TRPV4 to modulate TRPV4-based Ca^2+^ signalling (Yin et al., 2008). Rodent models have shown that TRPV4 on cortical astrocyte endfeet can evoke changes in intracellular astrocyte Ca^2+^ concentration, thereby modulating vascular tone and contributing to NVC (Dunn et al., 2013; Filosa et al., 2013). Rodent experiments support our observations by showing TRPV4 downregulation in streptozotocin-induced diabetes (Monaghan et al., 2015).

Retinal studies with streptozotocin-induced hyperglycemia in rodents have suggested that hyperplasia of Müller cells (retinal analogue of astrocytes) could lead to an increase in GFAP (Newman and Reichenbach, 1996; Rungger-Brandle et al., 2000). Astrocytic glutamate clearance is also impaired under high glucose conditions (Coleman et al., 2004), making neurons susceptible to depolarization, a possible cause of neurotoxicity. This could result in accumulation of glutamate in the extrasynaptic space, leading to recurrent neuronal depolarization. This is concordant with our observation of an increased number of spontaneous Ca^2+^ peaks in the glucose-treated larvae. Neuronal hyperexcitability and increased firing is known to be associated with seizures, commonly observed in diabetic patients (Martinez and Megias, 2009; Baviera et al., 2017). The various cellular markers shown here to be affected by glucose exposure could explain a predisposition of diabetics to seizures. Increased neuronal firing could also lead to abnormal and non-precise pre- and post-synaptic neuronal firing, causing defects in the synaptic plasticity mechanisms necessary for cognition and memory. Further exploration of this could help define the relationship between diabetes and cognitive defects.

The anatomical and molecular effects of glucose exposure on the NVU were associated with altered embryonic behaviour, with a reduction in preference for light, which is a measure of unconditioned anxiety and related disorders in rodents and zebrafish (Kulesskaya and Voikar, 2014; Kysil et al., 2017). Unconditioned anxiety is influenced by environmental, emotional and cognitive factors (Arrant et al., 2013). It is based on an approach/avoidance conflict between the drive to explore a novel area and an aversion to brightly lit/completely dark open spaces in adult/larval zebrafish, respectively (Bourin and Hascoet, 2003; Arrant et al., 2013). The approach/avoidance conflict is well studied in mammals and is known to have various neural substrates in the brain, such as in the limbic system, anterior cingulate cortex, ventral striatum and prefrontal cortex (Aupperle et al., 2015). Although zebrafish do not possess a cortex, their ventral and dorsal telencephalic area (Vd and Dm, respectively) are homologous to the mammalian amygdala and striatum (Maximino et al., 2013). Thus, impaired light/dark preference as described in the present study could imply an abnormal circuitry in the zebrafish Vd/Dm. Anatomical studies with zebrafish have shown that the Vd/Dm system projects to the optic tectum and hence any defects could also affect the optic tectum (Scott and Baier, 2009; Nevin et al., 2010).

Various studies have described larval zebrafish behavioural differences with anxiolytic or anxiogenic treatments (Egan et al., 2009; Richendrfer et al., 2012). However, this is the first study characterising the effect of hyperglycemia on geometrical and positional characteristics of zebrafish locomotion. Glucose exposure resulted in an increase in both exploration (as measured by the geometric features, eccentricity and MPDE) and thigmotaxis (as measured by an increase in the positional feature, MPDC). Various zebrafish studies have shown increased exploration and thigmotaxis with anxiogenic drug treatments (Egan et al., 2009; Blaser et al., 2010). This further points to the association of glucose exposure and diabetes to anxiety-related brain activation, which warrants further investigation. The use of behavioural assays, such as the novel-tank diving test (Levin et al., 2007), to assess anxiety-related freezing in glucose-exposed zebrafish would further strengthen the link between diabetes and anxiety.

Zebrafish show acclimatisation to novel surroundings, which could vary with developmental stage. Another future direction would be to explore whether glucose and/or SNP treatment could affect these acclimatization times (Kalueff et al., 2013).

In addition to being an effect of abnormal circuitry in the Vd/Dm, the observed impact of glucose exposure on the optic tectum may reflect visual dysfunctions (in the visual cortex and superior colliculus), which have been reported to occur before the onset of diabetes-associated retinopathy in mammalian studies (Honda et al., 1998; Ozsoy et al., 2012; Cui et al., 2014). Future studies with behaviour mapping to precise neural substrates are needed to further investigate the impact of glucose exposure on different brain circuitries.

Although human diabetes is a complex disorder and our zebrafish model examines only the effect of hyperglycemia, our findings broadly reproduce those in other cell-based and mammalian models (Williams et al., 1996; Li et al., 2002). Diabetes is well known to reduce vascular NO levels, and our work reproduces this. Although zebrafish are not known to possess eNOS (Syeda et al., 2013), our results strongly indicate vascular NO production. Previous studies have linked hyperglycemia and pharmacologically induced diabetes to reductions in cerebral blood flow (Dandona et al., 1978; Stevens et al., 2000). A recent study demonstrated rescuing effects of the NO donor SNP on hyperglycemia-induced neurovascular uncoupling (Chhabria et al., 2018). Using the same protocol as described by Chhabria et al. (2018) to induce hyperglycemia in larval zebrafish, we have now described multiple effects of hyperglycemia on cellular markers of the NVU, essential for regulation of CBF and on zebrafish behaviour.

This is the first study to demonstrate that SNP reverses the negative consequences of hyperglycemia on neurovascular anatomy and behaviour. The ability of SNP to prevent all the observed anatomical, molecular and behavioural effects of glucose exposure is exciting as it may represent a possible treatment for diabetes-associated neurovascular dysfunction. Future studies are needed to assess whether these effects of glucose exposure and SNP or other NO donors are seen in mammalian models or humans. NO donors are already widely used clinically for angina and heart failure, and are very cheap and off-patent. Therefore, if mammalian preclinical studies support our findings, clinical studies could rapidly be performed to examine the ability of NO donors to ameliorate or prevent the consequences of diabetes on neurological dysfunction.

## MATERIALS AND METHODS

### Transgenic zebrafish

All zebrafish studies were conducted in accordance with the Animals (Scientific Procedures) Act, 1986, United Kingdom and covered by Home Office Project Licence 70/8588 held by T.J.A.C. Reporting of experimental outcomes were in compliance with ARRIVE (Animal Research: Reporting In Vivo Experiments) guidelines (Kilkenny et al., 2010).

Maintenance of adult zebrafish was conducted according to previously described husbandry standard protocols at 28°C with a 14:10 h light:dark cycle (Lawrence, 2007).

The following zebrafish lines were used: *Tg(kdrl:HRAS-mCherry)^s91^^6^*, labelling EC membrane (Hogan et al., 2009); *Tg(klf2a:GFP)*, expressing GFP under control of the zebrafish *klf2a* promoter; *Tg(sm22ab:nls-mCherry)^sh480^*, labelling mural cells expressing a smooth muscle actin-binding protein; and *Tg(nbt:GCaMP3)*, which allows quantification of neuronal calcium levels (Bergmann et al., 2018).

### Glucose, mannitol and SNP treatment

Glucose, mannitol and SNP (Sigma) were prepared in E3 medium to final concentrations of 20 mM (glucose and mannitol) or 0.1 mM SNP (Chhabria et al., 2018). Zebrafish larvae for *in vivo* imaging or immunostaining were incubated in E3 medium containing glucose/mannitol from 4-9 dpf and SNP from 8-9 dpf (Chhabria et al., 2018).

### Assessment of NO reactivity in the cerebral vessels

Larvae exposed to glucose/mannitol with/without SNP as above were incubated with 2.5 µM DAF-FM in DMSO (1%) at 9 dpf for 3 h at 28°C in the incubator in the dark. Larvae were washed with glucose/mannitol solution to remove excess DAF-FM, and then imaged on the lightsheet fluorescent microscope (Zeiss Z1).

### Glutamine synthetase, GFAP and TRPV4 immunohistochemistry

IHC was used to observe and quantify changes in TRPV4 (OST00071W; Thermo Fisher Scientific) and the glial-specific markers GS (mab302; Millipore) and GFAP (zrf-1; Zebrafish International Resource Center). The protocol was adapted from Inoue and Wittbrodt (2011). Larvae for each different treatment (mannitol or glucose, with or without co-treatment with SNP) were fixed in 4% paraformaldehyde (PFA) overnight at 4°C followed by a 5 min wash with 1× PBS before resuspending in 100% methanol (MeOH) for storage at −20°C. Samples were rehydrated from MeOH with 3×10 min washes with phosphate buffer saline+0.1% Tween 20 (PBST), with gentle agitation on a rocker. Larvae were then suspended in 150 mM Tris-HCl (pH 9) for 5 min, followed by heating at 70°C for 15 min. Larvae then underwent 2×10 min washes with PBST, followed by 2× 5 min washes with distilled water (dH_2_O). Larvae were permeabilised using ice-cold acetone at −20°C for 20 min, followed by 2×5 min washes with dH_2_O, then equilibrated with 2×5 min washes in PBST.Subsequently, larvae were incubated in blocking buffer (B-buffer: 10% sheep serum, 0.8% Triton X-100 and 1% bovine serum albumin in PBST) for 3 h at 4°C. B-buffer was replaced with incubation buffer (I-buffer: 1% sheep serum, 0.8% Triton X-100 and 1% bovine serum albumin in PBST) containing the primary (1°) antibodies (Abs): 1:250 anti-GS (mouse monoclonal; mab302; Millipore), 1:100 anti-GFAP (mouse monoclonal; zrf-1; Zebrafish International Resource Center), 1:300 anti-TRPV4 (rabbit polyclonal; OST00071W; Thermo Fisher Scientific) and 1:1000 DAPI followed by incubation at 4°C for 3 days with gentle agitation on a rocker.

Residual 1° Abs were removed by 3× hourly washes in PBST [at room temperature (RT)]. Larvae then underwent 2×10 min washes with PBS+1% Triton X-100, followed by 2× hourly equilibration washes in PBS-TS (PBS+1% Triton X-100+10% sheep serum).

Larvae were incubated in I-buffer containing secondary antibodies (2° Abs): anti-rabbit, 488 nm (A11034, Invitrogen), anti-mouse, 647 nm (A28181, Invitrogen) and anti-mouse, 568 nm (A11004, Invitrogen), corresponding to respective 1° Abs, at 1:500 dilutions for 2.5 days, in the dark on a rocker at 4°C.

Prior to imaging on the lightsheet microscope, larvae were washed three times in PBS-TS (at RT), followed by two 1 h washes with PBST, each at RT. Larvae were mounted in 1% low-melting-point agarose (LMP; Sigma) and imaged for the glial patterning in the brain for different markers.

### Lightsheet fluorescent imaging

Lightsheet fluorescent microscopy (LSFM) was performed on 9 dpf larvae on a Zeiss Lightsheet Z.1 microscope. Larvae were minimally anaesthetised (using 4.2% v/v tricaine methanesulfonate) and embedded in 1% agarose in a glass capillary (inner diameter ∼1 mm) while imaging. We acquired 3D *z*-stacks with 800×600 pixels (1 pixel=0.6 µm) in *x-y* direction and a depth of 100 slices in *z* directions (slice thickness=1 µm).

For imaging spontaneous neuronal activity, we acquired time lapses of single ‘*z*’ plane optic tectum with our previous acquisition settings and frequency quantifications (Chhabria et al., 2018).

### Image analysis: *klf2a* quantification

Acquired 3D image stacks were converted to 2D maximum-intensity projections and the tectal vasculature was segmented out. Tectal vascular length was extracted (Chhabria et al., 2018). The segmented vasculature was used as a binary mask, followed by normalising the total intensity of the green channel (*klf2a:GFP*) in the optic tectum to the tectal vascular length.

### Image analysis: quantification of vascular mural cells

Acquired 3D image stacks were converted to 2D maximum-intensity projections followed by segmenting the *sm22ab^sh480^* nuclei (red channel) using intensity-based thresholding similar to the method of RBC segmentation described in Chhabria et al. (2018). Segmented cells in the optic tectum were enumerated (in a fixed vascular volume) using custom-written MATLAB scripts used for all treatment groups.

### Behavioural analysis

#### Light/dark preference

Analysis of light/dark preference of the larval zebrafish was designed on a similar principle to that of the adult zebrafish light/dark preference test (Blaser and Penalosa, 2011). A 12-well plate was modified by adhering three cellophane films (blue, green and yellow) to half of each well to create a ‘dark’ side that allowed the camera to track larvae movement by infrared (IR). Larvae from different treatment groups were placed on the light side of the well filled with 5 ml E3 (without Methylene Blue).

The plate was placed inside a Viewpoint Zebrabox system (1% light intensity) and the tracking protocol was built allocating dark and light regions of each well prior to the start of imaging (to get *x*,*y* coordinates of dark/light regions separately for analysis). Speed thresholds were set as high speed >6.4 mm/s, low speed 3.3-6.3 mm/s and inactive <3.3 mm/s. Total experimental duration was 1 h, inclusive of acclimatisation (recording started immediately after adding larvae to an individual well) to capture the overall behaviour. Analysis of parameters such as % time spent in the light, and % time spent in high and low speed locomotion were calculated in 1-min bins, and then averaged across the hour for each animal or averaged for 15-min intervals (Fig. S1). All the values of % time spent in low and high speed locomotion are normalised to % time spent in light and dark to avoid confounds due to differences in % time spent in light/dark for various treatment groups.

#### Automated locomotion analysis

**Fig. 8.**
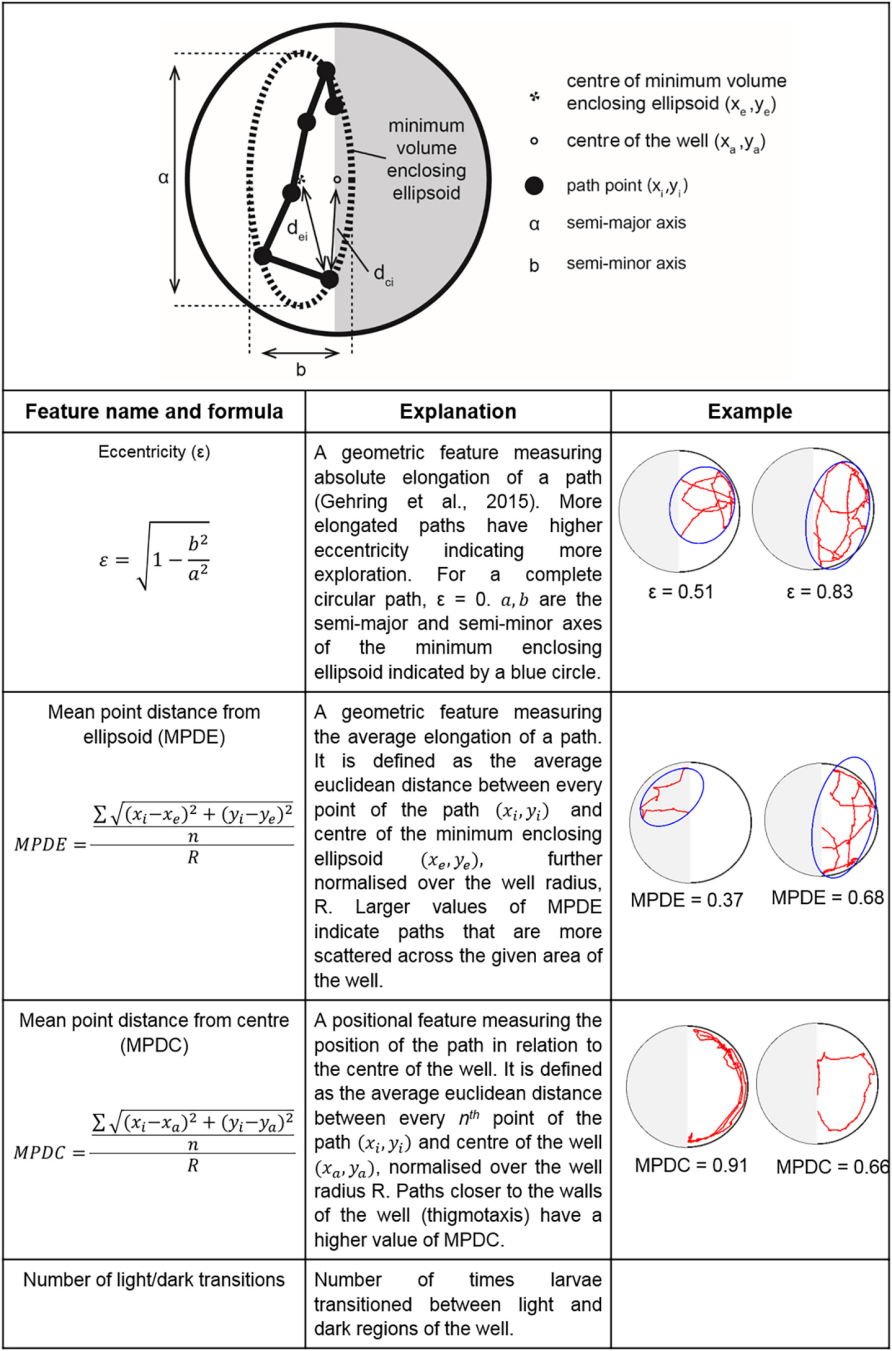
**Mathematical description of features calculated from the larval trajectories.** (Top) Schematic diagram of the well showing parameters calculated for various features. (Bottom) Table describing the formulae of calculated features for each coordinate of the trajectories. The minimum enclosing ellipsoid with centre (*x*_e_, *y*_e_) and major and minor axes of *a* and *b* is defined as the unique closed ellipse of smallest volume which enclose all points (*x*_i_, *y*_i_) of a path. 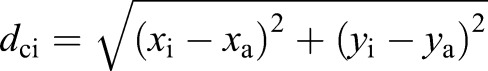 is the Euclidean distance of every point of the path to centre of the well (*x*_a_, *y*_a_).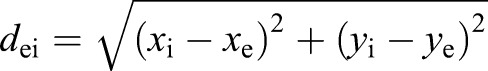 is the distance between the centre of minimum enclosing ellipsoid (*x*_e_, *y*_e_) and *i*^th^ point of the path. For each feature, two distinctive numeric examples are provided.

Using previous rodent-based methods developed for the Morris water maze (Wolfer and Lipp, 2000; Graziano et al., 2003; Gehring et al., 2015; Illouz et al., 2016; Vouros et al., 2018), we quantified four different features from the swimming trajectories of zebrafish. Coordinates of the swimming trajectories were extracted from the Viewpoint Zebrabox system and were segmented into smaller paths delimited by light/dark, followed by quantification of path features listed in Fig. 8. Small path segments, with lengths lower than 1st percentile of segments, generated as an artefact of light/dark transitions, were removed from further analysis. The path features eccentricity (ε) and MPDE quantify the spatial elongation of the locomotion trajectories and are used as measures of exploration in the field, while MPDC of the well represents thigmotaxis (preference of edge versus centre of well). Additionally, we quantified the number of transitions between light and dark areas (Fig. 8).

### Experimental design and statistical analysis

Experiments were designed using the National Centre for Replacement, Refinement, and Reduction of Animals in Research (NC3Rs) experimental design analysis (EDA) tool. GraphPad Prism (La Jolla) was used for statistical comparisons. Before performing statistical analysis, data were tested for normality using the Shapiro–Wilk test. All intergroup comparisons were performed using two-way ANOVA with post-hoc multiple-comparison tests (Sidak's test), where appropriate. *P*-values <0.05 were considered to be statistically significant. Data are shown as mean±standard error of mean (s.e.m.) unless specified. Imaging was performed unblinded to treatment allocation; data analysis was automated and thus not subject to operator bias. Randomisation of larvae to treatment group was not performed. All the data and MATLAB codes are available on request.

## Supplementary Material

Supplementary information
