# Supplementary material for: Sodium nitroprusside prevents the detrimental effects of glucose on the neurovascular unit and behaviour in zebrafish
Source: Dis Model Mech. 2019 Sep 25;12(9):dmm039867. doi: 10.1242/dmm.039867 (PMC6765192; doi:10.1242/dmm.039867)
Supplement: Supplementary information [file dmm-12-039867-s1.pdf]

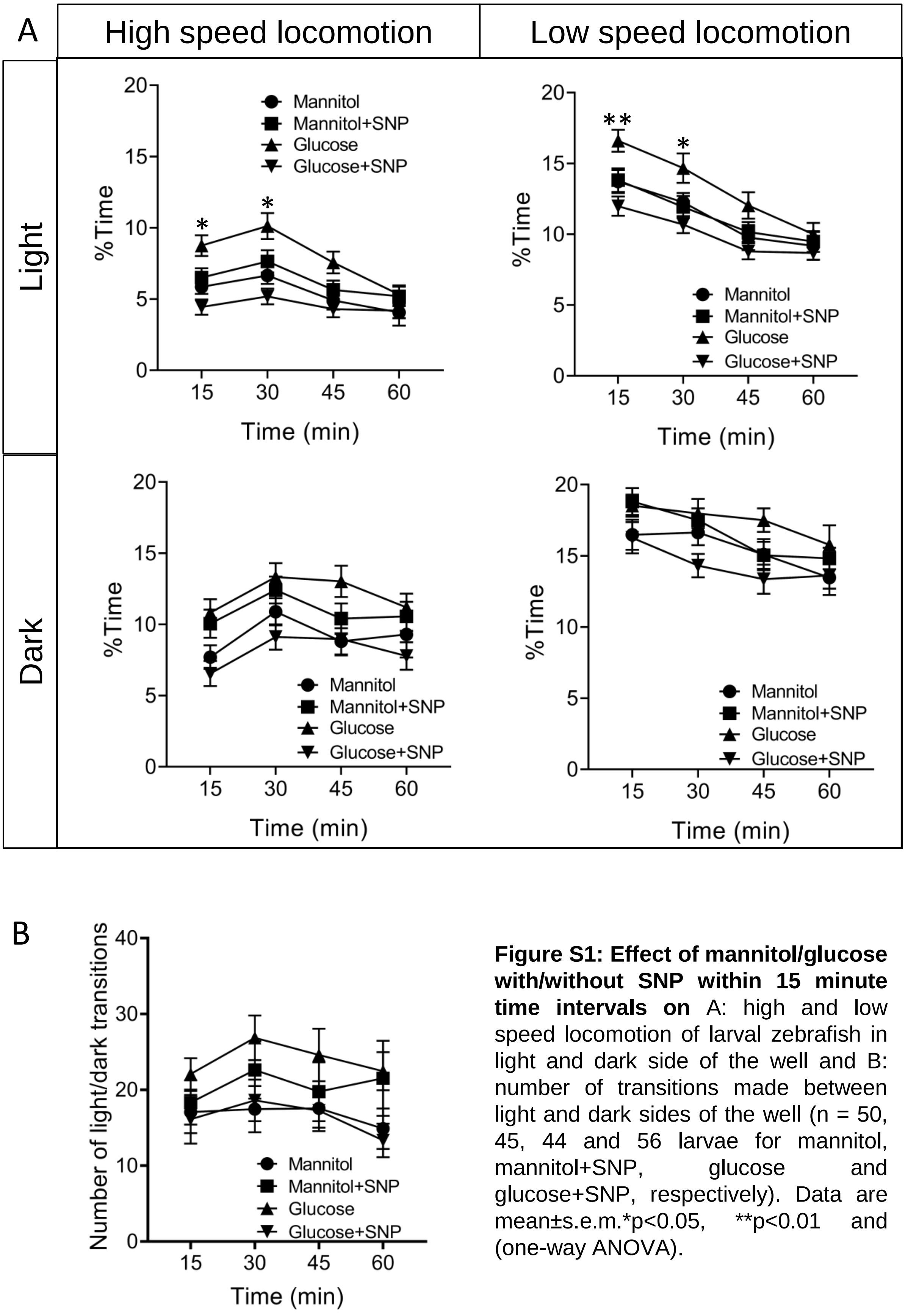

Figure S1: Effect of mannitol/glucose with/without SNP within 15 minute time intervals on A: high and low speed locomotion of larval zebrafish in light and dark side of the well and B: number of transitions made between light and dark sides of the well (n = 50, 45, 44 and 56 larvae for mannitol, mannitol+SNP, glucose and glucose+SNP, respectively). Data are mean±s.e.m.*$p<0.05$, **$p<0.01$ and (one-way ANOVA).

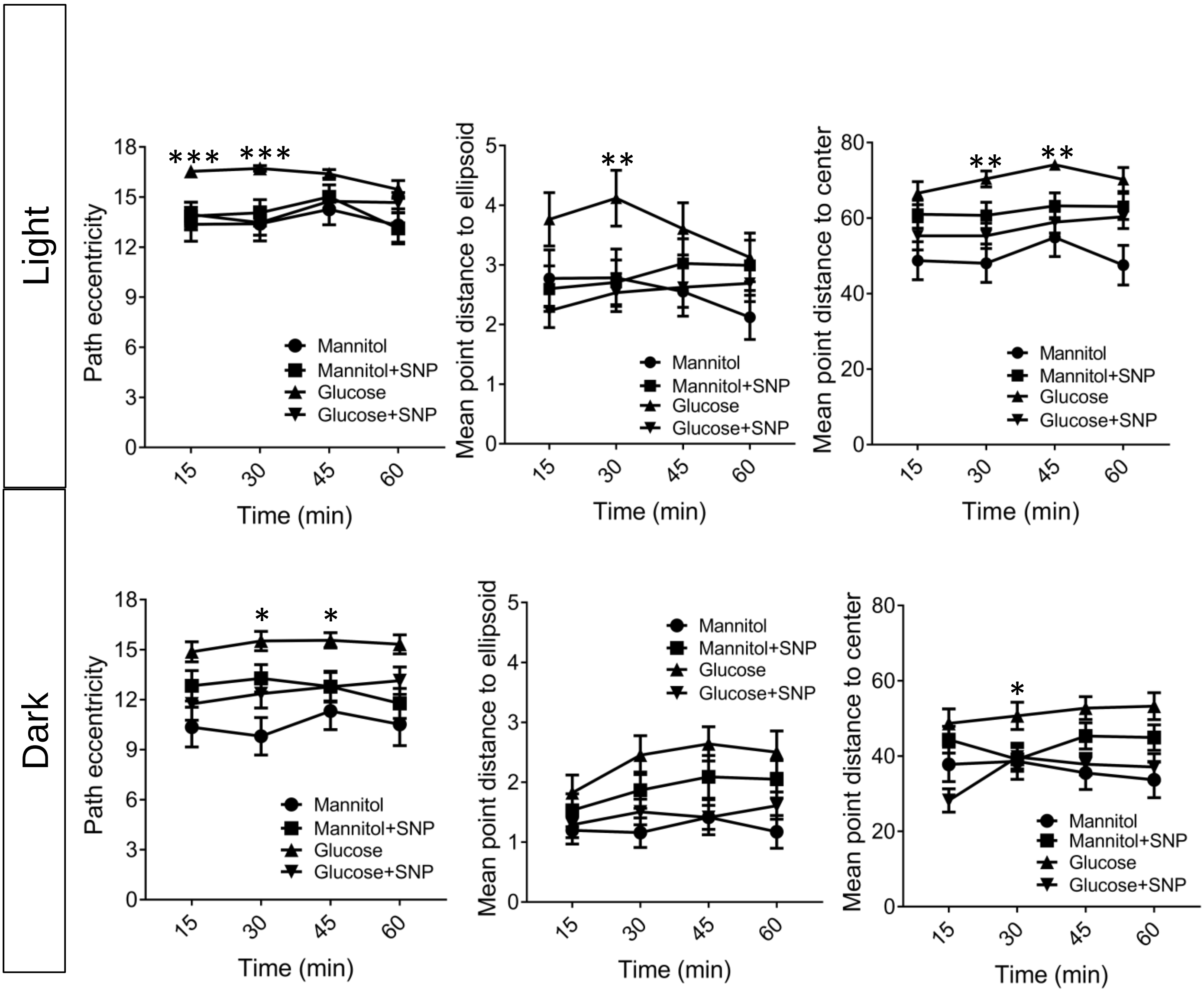

Figure S2: Effect of mannitol/glucose with/without SNP within 15 minute time intervals on various features of larval zebrafish behaviors; eccentricity, MPDE and MPDC in corresponding light and dark sides of the well (n = 50, 45, 44 and 56 larvae for mannitol, mannitol+SNP, glucose and glucose+SNP, respectively). Data are mean± s.e.m.*p<0.05, **p<0.01 and ***p<0.001 (one-way ANOVA)
